# Ignorance Isn't Bliss: We Must Close the Machine Learning Knowledge Gap in Pediatric Critical Care

**DOI:** 10.3389/fped.2022.864755

**Published:** 2022-05-10

**Authors:** Daniel Ehrmann, Vinyas Harish, Felipe Morgado, Laura Rosella, Alistair Johnson, Briseida Mema, Mjaye Mazwi

**Affiliations:** ^1^Department of Critical Care Medicine, Hospital for Sick Children, Toronto, ON, Canada; ^2^Temerty Centre for Artificial Intelligence Research and Education in Medicine, University of Toronto, Toronto, ON, Canada; ^3^MD/PhD Program, Temerty Faculty of Medicine, University of Toronto, Toronto, ON, Canada; ^4^Institute for Health Policy, Management and Evaluation, Dalla Lana School of Public Health, University of Toronto, Toronto, ON, Canada; ^5^Department of Medical Biophysics, Temerty Faculty of Medicine, University of Toronto, Toronto, ON, Canada; ^6^Program in Child Health Evaluative Sciences, The Hospital for Sick Children, Toronto, ON, Canada

**Keywords:** artificial intelligence, machine learning, pediatric critical care medicine, medical education, learning curricula

## Abstract

Pediatric intensivists are bombarded with more patient data than ever before. Integration and interpretation of data from patient monitors and the electronic health record (EHR) can be cognitively expensive in a manner that results in delayed or suboptimal medical decision making and patient harm. Machine learning (ML) can be used to facilitate insights from healthcare data and has been successfully applied to pediatric critical care data with that intent. However, many pediatric critical care medicine (PCCM) trainees and clinicians lack an understanding of foundational ML principles. This presents a major problem for the field. We outline the reasons why in this perspective and provide a roadmap for competency-based ML education for PCCM trainees and other stakeholders.

## Introduction

Pediatric intensivists are bombarded with more patient data than ever before. The density and complexity of data generated from patients, their monitoring devices, and electronic health records (EHR) pose significant cognitive challenges. Clinicians are required to integrate data from a variety of sources to inform medical decision-making, which is further challenged by high stakes, time-sensitivity, uncertainty, missing data, and organizational limitations (1, 2).

These constraints make critical care environments a compelling use case for artificial intelligence (AI) in medicine. AI is an umbrella term that contains multiple techniques and approaches. Modern advances in AI have largely been driven by machine learning (ML) methods such as supervised, unsupervised, deep, and reinforcement learning (3). ML has the potential to decrease cognitive load and enhance decision making at the point of care. Additionally, ML may be uniquely suited to analyzing the heterogeneous data generated during care and quantifying the complex determinants of the behavior of critically ill patients. Techniques, expectations, and infrastructures for developing and utilizing ML have matured, and there are many examples of ML algorithms published in the critically ill adult (4–8) and pediatric (9–16) literature that robustly predict morbidities and mortality.

However, these algorithms are at risk of being deployed in an environment where many intended end-users currently lack a basic understanding of how they work (17–20). We argue that the ML education gap in pediatric critical care medicine (PCCM) presents a major problem for the field because it may contribute to either *distrust* or *blind trust* of ML, both of which may harm patients.

## Why Lack of ML Education is a Problem for PCCM

Clinician distrust of ML is inversely associated with clinician engagement with the ML tool. Distrustful, disengaged clinicians are less likely to use even well-performing ML (21), limiting potential benefits to patients. Distrust may also manifest as missed opportunities to demystify the technology for trainees, recruit clinician champions for future ML projects, and realize the return on institutional and/or extramural investment. Distrust can therefore be enormously costly—in both non-monetary and monetary terms—and efforts to combat clinician distrust in ML through extensive pre-integration education have been successfully employed in both adult (22) and pediatric critical care (23).

Conversely, blind trust of ML is also problematic for PCCM. Humans are prone to automation bias whereby automated decisions are implicitly trusted, especially when end-users poorly understand the subject matter (24). Automation bias has been reported in the ML literature, especially among inexperienced end-users (25). In PCCM, blind trust of ML has the potential to harm patients. Clinicians may make flawed decisions when inappropriately using algorithms developed using biased training datasets (26). Furthermore, algorithms may degrade in performance over time (27) and across different care settings (28). These phenomena may be more prevalent in ML developed from relatively small training datasets (12), as in PCCM. Pediatric intensivists must be able to critically appraise ML literature and any ML-based tool. Identifying strengths and weaknesses of any potential ML intervention is vital to its proper application at the bedside, and critically ill pediatric patients deserve the same rigor applied to ML as other important topics in PCCM.

## Closing the Gap: A Proposed ML Curriculum for PCCM Trainees and Other Stakeholders

After acknowledging that a ML knowledge gap exists in PCCM, we must make concerted efforts to close it for the benefit of our patients. Understanding foundational principles of ML will be required to effectively interact with many applications of ML, including diagnostic and therapeutic decision support systems (e.g., disease risk prediction models, treatment recommender systems, etc.). Published ML curricula for medical students (29, 30) and medical (31, 32) and surgical (33) subspecialities converge on several key domains such as the “critical appraisal of AI systems” and “ethical and legal implications” (34). We propose below an ML curriculum for PCCM trainees and stakeholders based on similar domains, but with key adaptations for clinicians caring for critically ill children where appropriate.

We used Kern's six step approach for curriculum development (35) as a guiding framework for our ML in PCCM curriculum (Table 1). After identifying the PCCM education gap problem above (Kern's Step 1), our group of experts in PCCM, medical education, and ML identified high-priority curricular needs (Kern's Step 2) based on previous literature (30) and group consensus using modified Delphi methodology (36). We determined specific curriculum objectives (Kern's Step 3), which were operationalized into measurable, enabling competencies. Competencies were designed to be checked “yes, achieved” or “no, not achieved” at the competition of the curriculum and/or PCCM training. We suggest educational strategies (Kern's Step 4) to achieve specific competencies.

**Table 1:** Proposed PCCM ML curriculum.

**Curriculum objective**	**Enabling competencies**	**Possible educational strategies**
A. Foundational ML concepts from development to deployment	A1. Describe and identify major classes of machine learning (e.g., supervised/unsupervised learning, deep learning, reinforcement learning) and the phases of applying ML in critical care settings from development through deployment A2. Describe key differences in data sources and structure required to build different classes of ML A3. Recognize limitations of training data common to pediatrics and PCCM (e.g., data sparsity) and possible mitigation strategies A4. Explain methodological concepts integral to ML model evaluation (e.g., validation, bias, variance, etc.) and performance (e.g., sensitivity, specificity, positive predictive value, precision, receiver operating characteristic curves, F-1 score, etc.) A5. Demonstrate appropriate application of different ML techniques to specific use cases in PCCM A6. Gain a foundational understanding of the “human” factors relevant to using ML at the bedside (e.g., cognitive biases, cognitive load, trustworthiness, uncertainty, explainability, etc.) A7. Learn specific strategies to discuss the results of ML systems with pediatric patients (when applicable) and families	• Asynchronous online module with subsequent small group discussion (Competencies A1 and A4) • Interprofessional discussion and case-based learning with data scientists and engineers (Competencies A2, A3, and A5) • Simulation (Competencies A5, A6 and A7)
B. ML Ethical and legal considerations in clinical practice	B1. Explain the issues of bias and inequity in ML algorithms, including its potential etiologies and implications using published examples B2. Understand core concepts of data privacy and how they relate to building and using ML B3. Explain the challenges associated with using ML for shared decision making in PCCM with families and pediatric patients B4. Identify sources of liability when using ML outputs to guide decision-making and how to navigate liability with families and regulators	• Bioethics case-based discussion (Competency B1) • Case-based didactic learning with clinicians and administrators (Competencies B2 and B4) • Simulation (Competency B3)
C. Proper usage of EHR and biomedical data	C1. Understand broadly how EHR data is used to build ML, including key benefits and limitations to the approach (e.g., data missingness, data incorrectness, lack of granularity, etc.) and how limitations are typically managed C2. Understand the limitations of applying ML to the common pathologies of PCCM (e.g., patient heterogeneity, age-specific variance, etc.) and strategies to mitigate limitations when possible C3. Explain some future directions of biomedical data and ML, including novel sources of healthcare data in critical care (e.g., imaging, genetic data, inflammatory profiles, unstructured/text data, wearable data, etc.)	• Interprofessional discussion with data scientists, computer scientists, and health informatics specialists (Competencies C1 and C2) • Asynchronous online module (Competency C3)
D. Critical appraisal of ML systems	D1. Appraise ML tools/literature based on evidence-based medicine principles (e.g., internal validity, generalizability, risk of bias) D2. Understand the core components of reporting guidelines for ML and its prospective evaluation	• Case-based discussion with ML clinician champions and researchers (Competency D1) • Asynchronous online module with subsequent small group discussion (Competency D2)

**Figure 1:**
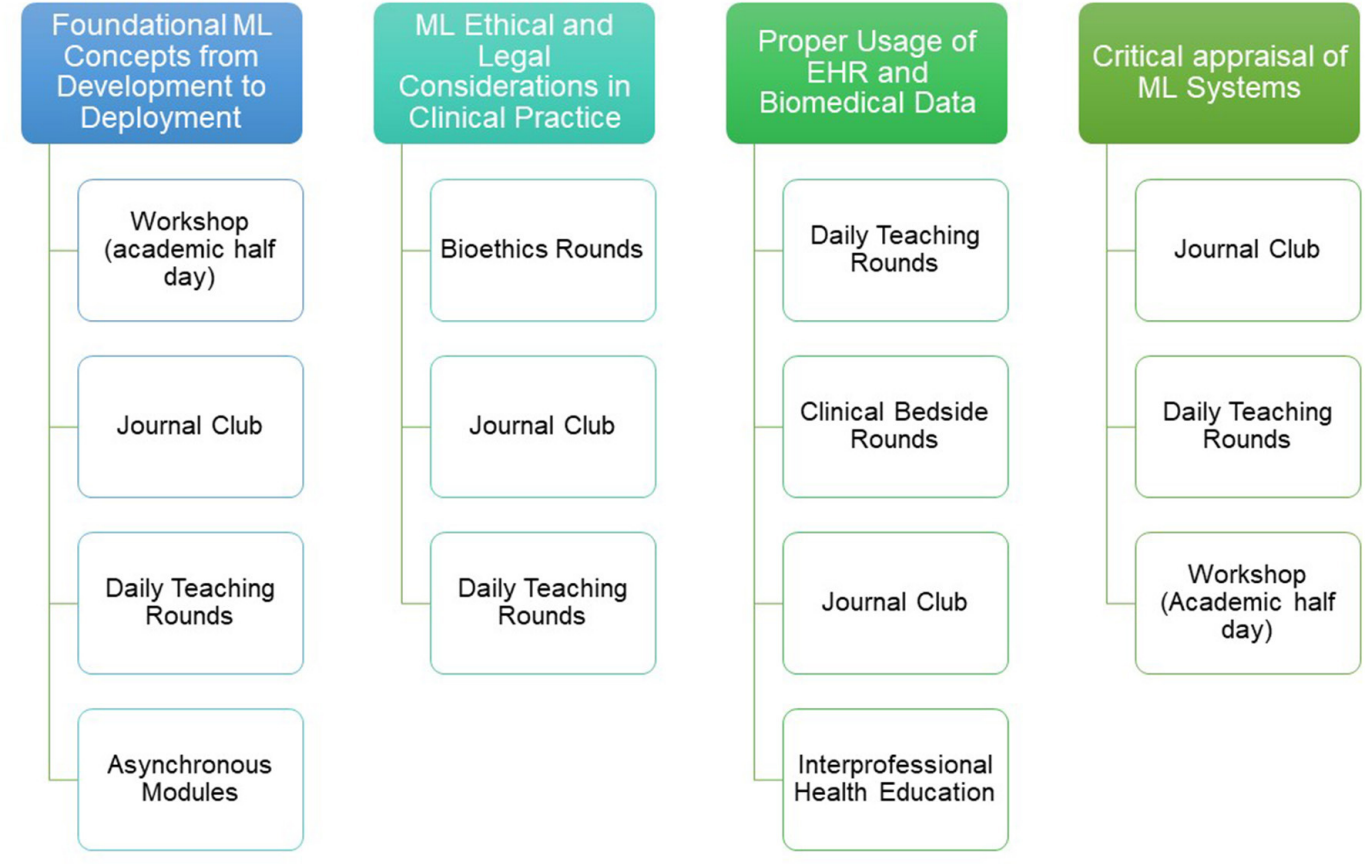
One potential roadmap for leveraging existing curricular implementation resources common to many PCCM training programs. Resources are divided by the ML in PCCM curriculum objectives.

Step 5 of Kern's approach relates to implementation, which is the practical deployment of the education strategies listed above within the context of PCCM training resources and modalities. Many of the forums/methods necessary to institute the ML in PCCM curriculum already exist in many programs, thereby increasing the feasibility of delivery. We outline implementation resources common to many PCCM training programs, organized by curriculum objective, in Figure 1. Implementation of the curriculum may be more challenging in institutions that lack these resources. Shared access to materials that can be delivered virtually (e.g., freely accessible online modules, ML conferences/webinars, discussion with computer scientists via video conference, etc.) may increase the feasibility of curriculum implementation at less resourced centers. Curriculum champions at early adopting centers can also provide mentorship and promote faculty development at centers that have the desire to implement the curriculum but lack ML experience or expertise.

The final step of Kern's approach relates to evaluation and assessment (35). We recommend a multifaceted approach. Traditional pre-post assessments using the Kirkpatrick outcomes hierarchy (37) can collect objective data such as knowledge of core ML concepts and subjective data such as trainee confidence applying those concepts. These assessments should be combined with open-ended discussions with key stakeholders (i.e., educational and institutional leaders, clinical faculty, trainees, interprofessional team/allied health members, course teachers, etc.) regarding key outcomes of interest. This multifaceted approach acknowledges the known limitations of pre-post assessments in richly understanding how a curriculum impacts learners. Evaluations should be repeated longitudinally to measure retention and identify new high-yield curricular objectives for PCCM that may arise in the fast-changing field of ML.

## Conclusions

The promise of ML to improve medical decision making and patient outcomes is tempered by an incomplete understanding of the technology in PCCM. This education gap presents a major problem for the field because trainees and key stakeholders are at risk for developing distrust or blind trust of ML, which may negatively impact patients. However, this problem also presents an opportunity to effectively close the education gap by instituting an ML in PCCM curriculum. Our multidisciplinary group is the first to present such a curriculum in this perspective, focusing on key high-yield objectives, measurable enabling competencies, and suggested educational strategies that can utilize existing resources common to many PCCM training programs. We hope to empower PCCM trainees and stakeholders with the skills necessary to rigorously evaluate ML and harness its potential to benefit patients.

## Data Availability Statement

The original contributions presented in the study are included in the article/supplementary material, further inquiries can be directed to the corresponding author/s.

## Author Contributions

DE, VH, and FM: literature search, background and rationale, writing all or part of the manuscript, critical revision of the manuscript, and editing of the manuscript. LR, AJ, and BM: critical revision of the manuscript and editing of the manuscript. MM: background and rationale, critical revision of the manuscript, and editing of the manuscript. All authors agree to be accountable for the content of the work. All authors contributed to the article and approved the submitted version.

## Funding

VH was supported through an Ontario Graduate Scholarship, Canadian Institutes of Health Research Banting and Best Master's and Doctoral Awards, and Vector Institute Postgraduate Fellowship. LR was supported through a Canada Research Chair in Population Health Analytics (950-230702).

## Conflict of Interest

The authors declare that the research was conducted in the absence of any commercial or financial relationships that could be construed as a potential conflict of interest.

## Publisher's Note

All claims expressed in this article are solely those of the authors and do not necessarily represent those of their affiliated organizations, or those of the publisher, the editors and the reviewers. Any product that may be evaluated in this article, or claim that may be made by its manufacturer, is not guaranteed or endorsed by the publisher.
